# Evidence for a multicomponent hierarchical representation of dual tasks

**DOI:** 10.3758/s13421-020-01097-3

**Published:** 2020-09-28

**Authors:** Patricia Hirsch, Clara Roesch, Iring Koch

**Affiliations:** Cognitive and Experimental Psychology, Institute of Psychology, RWTH Aachen University, Jägerstr. 17-19, D-52066 Aachen, Germany

**Keywords:** Task organization, Dual tasks, Global level of processing, Task pairs

## Abstract

Recent dual-task studies observed worse performance in task-pair switches than in task-pair repetitions and interpreted these task-pair switch costs as evidence that the identity of the two individual tasks performed within a dual task is jointly represented in a single mental representation, termed “task-pair set.” In the present study, we conducted two experiments to examine (a) whether task-pair switch costs are due to switching cues or/and task pairs and (b) at which time task-pair sets are activated during dual-task processing. In Experiment 1, we used two cues per task-pair and found typical dual-task interference, indicating that performance in the individual tasks performed within the dual task deteriorates as a function of increased temporal task overlap. Moreover, we observed cue switch costs, possibly reflecting perceptual cue priming. Importantly, there were also task-pair switch costs that occur even when controlling for cue switching. This suggests that task-pair switching per se produces a performance cost that cannot be reduced to costs of cue switching. In Experiment 2, we employed a go/no-go-like manipulation and observed task-pair switch costs after no-go trials where subjects prepared for a task-pair, but did not perform it. This indicates that task-pair sets are activated before performing a dual task. Together, the findings of the present study provide further evidence for a multicomponent hierarchical representation consisting of a task-pair set organized at a hierarchically higher level than the task sets of the individual tasks performed within a dual task.

Making notes while following a research talk, or having a conversation with passengers while driving a car—these are only a few examples that highlight how often we engage in performing two tasks in a temporal overlap and hence in dual-tasking. Performance in such dual-task situations has been extensively studied to gain insights into the fundamental aspects of the cognitive architecture and the basic principles of human information processing (see, e.g., Koch, Poljac, Müller, & Kiesel, 2018, for a review). In general, two lines of dual-task research can be distinguished.

The first, traditional and long-established, research line deals with the *local level of dual-task processing*. This level focuses on cognitive processes and information related to one of the two individual tasks performed within a dual task. Within the scope of this research line, it has been shown, for instance, that dual-tasking ordinarily results in performance costs which reflect worse performance in a specific task in situations with dual-task requirements than in situations with no or fewer dual-task requirements (see Pashler, 1994, for a review).

The second research line, which has recently gained more attention, addresses the *global level of dual-task processing*. This level refers to cognitive processes and information that are related not only to one of the two individual tasks performed within a dual task but to both individual tasks at the same time. In this context, first evidence has emerged that the identity of the two individual tasks performed within a dual task is jointly represented in a single mental representation, termed “task-pair set.” This indicates that the two individual tasks of a dual task represent the subtasks of a more complex single task, such as starting a car with the overlapping subtasks of releasing a clutch and pressing the gas pedal (e.g., Hirsch, Nolden, & Koch, 2017; Hirsch, Nolden, Phillip, & Koch, 2018).

The present study ties in with these findings. In Experiment 1, we aimed to exclude an alternative explanation for the role of task-pair sets in dual-tasking, and in Experiment 2, we went beyond the previous studies by addressing the novel theoretical question of the point in time when task-pair sets are activated during dual-task processing.

## The local level of dual-task processing

In the context of the research line on the local level of dual-tasking, the cognitive processing of one of the two individual tasks performed within a dual task is analyzed. The cognitive processes can, for instance, operate on the mental representation of an individual task, referred to as “task set.” There are multiple proposals regarding what exactly a task set is (e.g., Mayr & Keele, 2000; Meiran, 2000; Rogers & Monsell, 1995; see e.g., Kiesel et al., 2010; Vandierendonck, Liefooghe, & Verbruggen, 2010, for an overview). Generally, it is assumed that a task set represents different components, including the task-relevant stimulus-set, response-set, and set of stimulus–response mappings. These components guide cognitive processing from stimulus encoding to responding, thereby assuring the correct execution of a task and the achievement of the task goal. Task sets can also be defined more formally as a set of parameters that are required to program a model to correctly perform a task (e.g., Logan & Gordon 2001; Schneider & Logan, 2005).

### The PRP paradigm

Cognitive processing at the local level of dual-tasking is often examined with the psychological refractory period (PRP) paradigm (e.g., Welford, 1952; see, e.g., Koch et al., 2018; Pashler, 1994, for reviews). In this paradigm, subjects perform two independent tasks, Task 1 (T1) and Task 2 (T2), which are linked to separate stimuli, Stimulus 1 (S1) and Stimulus 2 (S2), and to separate responses (R1 and R2). The presentation of S1 and S2 is separated by a varying stimulus-onset asynchrony (i.e., SOA, which is the time interval between the onsets of S1 and S2), and subjects are typically instructed to respond first to T1 and then to T2.

A typical finding with this paradigm is that T1 performance is unaffected by SOA variations (i.e., no SOA effect), whereas T2 performance deteriorates with decreasing SOA—hence, an increasing temporal overlap between the processing of T1 and T2 (see, e.g., Strobach, Schütz, & Schubert, 2015, for a review on the effects on T1 performance). The T2 performance deterioration as a function of SOA (i.e., SOA effect in T2) is referred to as the PRP effect (see, e.g., Pashler, 1994, for a review).

### Accounts of the PRP effect

Traditional accounts of dual-task interference attribute the PRP effect to an information processing bottleneck at the stage of decision and response selection (see, e.g., Fischer & Plessow, 2015; Koch et al., 2018, for reviews). This bottleneck is assumed to occur either mainly because of structural characteristics inherent in the cognitive architecture and/or due to more “active” cognitive control mechanisms strategically optimizing the temporal coordination of the simultaneous processing of multiple tasks.

According to accounts of the structural perspective, the processing of each task comprises three serial processing stages—namely, perceptual processes, response selection, and motor processes. Whereas responses cannot be selected for two tasks at once, constituting a processing bottleneck, all other stages can proceed for T1 and T2 in parallel. For which task responses are selected at first is determined passively based on a “first-come-first-served” basis. Thus, with short SOA, perceptual processing starts and ends almost simultaneously for T1 and T2, so that T2 processing is queued until the T1 response has been selected. In contrast, with long SOA, this T2 postponement is reduced because perceptual processing in T2 can proceed during response selection for T1.

In contrast to structural models, models hypothesizing the involvement of cognitive control in the occurrence of the PRP effect postulate that the PRP effect results from a strategic decision for sequential response selection because sequential response selection prevents unwanted response reversals (e.g., Logan & Gordon, 2001; Meyer & Kieras, 1997a). For instance, in the executive-process/interactive-control (EPIC) architecture (Meyer & Kieras, 1997a, 1997b), an executive process rule set coordinates T1 and T2 processing. To this end, it specifies the point in time when T1 and T2 processing can start, lock-out points in T2, where T2 processing is suspended, and lock-in events in T1, which signal T2 processing to continue. In PRP settings, T2 processing can be deferred to ensure that the response for T1 is produced before the response for T2 by setting the lock-in events, for example, after T1 response selection.

Importantly, models on the PRP effect have inspired a substantial body of empirical and theoretical research, resulting in a venerable research tradition. Yet, despite this fruitful research perspective, it is important to acknowledge that, in addition to the local level of dual-tasking, there is a global level of dual-task processing.

## The global level of dual-task processing

In contrast to the research line on the local level of dual-tasking, where the focus lies exclusively on one of the two individual tasks performed in a dual task, the research line on the global level of dual-tasking focuses on cognitive processes and information related to both T1 and T2 at the same time. Most studies on the global level of dual-task processing have so far examined order control in dual tasks.

### Order-switching logic

PRP studies on order control predominantly used the order-switching logic (e.g., Kübler, Reimer, Strobach, & Schubert, 2018; Kübler & Schubert, 2017; Kübler, Soutschek, & Schubert, 2019; Luria & Meiran, 2003, 2006; Szameitat, Lepsien, von Cramon, Sterr, & Schubert, 2005; Szameitat, Schubert, Müller, & von Cramon, 2002; see also De Jong, 1995). According to this logic, two tasks (e.g., A and B) are combined to two subtask orders (i.e., Order 1: A as T1 and B as T2 [i.e., AB]; Order 2: BA). The sequence of the subtask orders is varied, resulting in order-switch trials and order-repetition trials. In order-switch trials, the subtask order differs from that in trial *n* − 1 (e.g., Order 2 ➔ Order 1). In order repetition trials, the subtask order is the same as that in trial *n* − 1 (e.g., Order 1 ➔ Order 1).

Typically, T1 and T2 performance is worse in order-switch trials than in order-repetition trials, leading to order-switch costs (see Schubert, 2008, for a review). Note that the order-switch cost occurs even though there is a switch at the local level of T2 in trial *n − 1* and T1 in trial *n* in order repetition trials (e.g., AB ➔ AB), and a task repetition between T2 in trial *n − 1* and T1 in trial *n* in order-switch trials (e.g., AB ➔ BA). Studies in the general task-switching domain showed that performance is impaired in task switches as compared with task repetitions (see, e.g., Kiesel et al. 2010; Koch, Gade, Schuch, & Philipp, 2010; Koch et al., 2018; Monsell, 2003; Vandierendonck et al., 2010, for reviews). Thus, in studies using the order-switching logic, performance should have been worse in order-repetition trials with a task switch between T2 and T1 across trials than order-switch trials with a task repetition between T2 and T1 across trials.

The order-switch cost is assumed to indicate that the order of the two tasks in a dual task has to be specified and that order information is cognitively represented as an “order set” (e.g., Luria & Meiran, 2003). The order set contains information about both T1 and T2 and is therefore related to the global level of dual-tasking.

### Task-pair switching logic

Further evidence for cognitive processing at the global level of dual-tasking comes from studies on hierarchical task organization in dual tasks by Hirsch and colleagues (Hirsch et al., 2017, 2018). They developed a novel empirical approach, called “task-pair switching logic”. According to this logic, three tasks (e.g., A, B, and C) are combined to two task pairs (i.e., PRP trials) with a constant T1 and a varying T2 or vice versa (e.g., Task-Pair 1 with C as T1 and A as T2 [i.e., CA]; Task-Pair 2: CB). To assess the aftereffects of the cognitive processing in a given trial on the performance in the following trial, the task-pair sequence is manipulated on a trial-by-trial basis, and a cue is used at the beginning of each trial to indicate the task pair to be performed. The task-pair sequence variation leads to task-pair switch trials and task-pair repetitions trials. In task-pair switch trials, the task pair in a given trial differs from that in the previous trials (e.g., Task-Pair 2 ➔ Task-Pair 1), whereas in task-pair repetition trials, a task-pair is repeated across two consecutive trials (e.g., Task-Pair 1 ➔ Task-Pair 1).

Performance is typically worse in task-pair switch trials than in task-pair repetition trials, leading to task-pair switch costs in both T1 and T2. The task-pair switch cost is a robust effect that has been found with distinct task types for T1 (i.e., T1 with speeded manual responses or deferred nonspeeded vocal responses; e.g., Hirsch et al., 2017; Koch & Rumiati, 2006), varying extent of overlap in the response sets of T1 and T2 (i.e., conceptual and physical response-set overlap), and several numbers of task pairs (i.e., two and three; Hirsch et al., 2017, 2018).

### Accounts of task-pair switch costs

Note that there is a switch between T2 of the task-pair in trial *n −* 1 and T1 of the task-pair in trial *n* both in task-pair switch trials (e.g., Task-Pair 1 with CA in trial *n −* 1 and Task-Pair 2 with CB in trial *n*) and in task-pair repetition trials (Task-Pair 2 with CB in trial *n* − 1 and Task-Pair 2 with CB in trial *n*; see column T2–T1 switch in Table 1). Consequently, task-pair switch costs in T1 cannot be attributed to switching at the local level of T2 and T1.

**Table 1:** Sequences at the global task-pair level (i.e., whether there is a switch at the level of task pairs and task-pair cues) and the local subtask level (i.e., whether T1 and T2 switch across task pairs and whether there is a switch between T2 of trial *n −* 1 and T1 of trial *n*) in previous task-pair switching studies and in Experiment 1 of the present study

Task-pair transition	Global task-pair switch	Task-pair cue switch	Local T1–T1 switch	Local T2–T2 switch	Local T2–T1 switch
Previous task-pair switching studies					
CA ➔ CA	*x*	*x*	*x*	*x*	✓
CB ➔ CB	*x*	*x*	*x*	*x*	✓
CA ➔ CB	✓	✓	*x*	✓	✓
CB ➔ CA	✓	✓	*x*	✓	✓
Present study (Experiment 1)					
CA ➔ CA	*x*	✓	*x*	*x*	✓
CB ➔ CB	*x*	✓	*x*	*x*	✓
CA ➔ CB	✓	✓	*x*	✓	✓
CB ➔ CA	✓	✓	*x*	✓	✓

Task-pair switch costs have, therefore, been interpreted as providing evidence that information about the identity of T1 and T2 was activated in the previous trial, and that this activation persisted into the next trial. The persisting activation hampers performance when another task pair is performed and facilitates it when the same task pair is performed again. Hence, task-pair switch costs indicate that the identity of T1 and T2 is jointly represented in a single mental representation, referred to as task-pair set. Following this rationale, T1 and T2, which are performed in a temporal overlap within a dual task, represent the subtasks of a more complex hierarchical higher-order representation. Overall, there is, thus, first evidence for a multicomponent mental representation of a dual task, including a task-pair set and the T1 and T2 task sets.

However, Hirsch et al. (2018) state that only task-pair switch costs in T1 should be interpreted as evidence for such a multicomponent mental representation of a dual task and that the task-pair switch cost in T2 has to be interpreted with caution. This is because most models on dual-task interference assume sequential response selection, leading to the situation that each prolongation of perceptual processing and/or response selection in T1 results in queuing of T2 response selection for the same time. Thus, task-pair switch costs in T2 might simply propagate from T1 to T2. In this case, the T2 task-pair switch cost would not reflect performance costs arising in T2 itself due to task-pair switching.

### The present study

Hirsch and colleagues (2017, 2018) reported novel findings relating to task organization in dual tasks. However, since they used only one cue per task pair, task-pair switches were always accompanied by a cue switch, whereas task-pair repetitions came along with a cue repetition (see column Task-Pair Cue Switch in Table 1). Hence, it is not clear to which degree task-pair switching per se produces task-pair switch costs or whether this performance cost could also be partly due to repetition priming at the level of cue processing instead of switching task pairs.

Moreover, the point in time when task-pair sets are activated during dual-tasking remains unclear. First, a task-pair set might have to be activated *before* starting to perform a dual task, and second, the task-pair set might be formed as an episodic representation of the previous trial, and hence, be only available *after* performing a dual task. Moreover, it is conceivable that both possibilities contribute to task-pair switch costs.

The present study focuses on both these open questions. In Experiment 1, we examined how cue switching contributes to task-pair switch costs, thereby excluding cue switching as alternative explanation for task-pair switch costs. In Experiment 2, we went beyond the findings of the previous task-pair switching studies and investigated the point in time when task-pair sets are activated.

## Experiment 1

The goal of Experiment 1 was to examine how cue switching contributes to the task-pair switch cost observed in previous studies using one cue per task pair. With such a 1:1 mapping of cues to task pairs, it cannot be ruled out that task-pair switch costs emerge because of switching cues instead of task pairs. To disentangle the effects of cue switching from the effects of task-pair switching and to determine their relative contributions to the task-pair switch cost assessed with the 1:1 cue to task-pair mapping, we have conducted a task-pair switching study with two cues per task-pair (see, e.g., Arrington, Logan, & Schneider, 2007; Logan & Bundesen, 2003; Mayr & Kliegl, 2003, for this procedure in the task-switching domain; see Jost, De Baene, Koch, & Brass, 2013, for a review). This 2:1 cues to task-pair mapping resulted in task-pair switch trials with a cue switch, task-pair repetition trials with a cue switch, and task-pair repetition trials with a cue repetition.

By contrasting performance across task-pair switch trials and task-pair repetition trials with a cue switch, task-pair switching can be examined independently from cue switching and, thus, a “pure” task-pair switch cost can be assessed. By comparing performance across task-pair repetition trials with a cue switch and with a cue repetition, the effect of cue switching, termed cue-switch cost, is assessed separately from task-pair switching.

In the general task-switching domain, studies using the 2:1 mapping differ with respect to whether they reported, in addition to cue-switch costs, substantial “pure” task-switch costs (e.g., Logan & Bundesen, 2003; Mayr & Kliegl, 2003), even though most studies reported clear task-switching costs. To account for these conflicting findings, different task-switching models have been put forward.

Models that are based on the finding that task switching produces cue-switch costs, but no “pure” task-switch costs, postulate that the cued task-switching paradigm does not require task switching, and hence endogenous task-set reconfiguration. Rather, task-switch costs in this paradigm would reflect cue-encoding benefits due to lower-level priming processes in task repetition trials. This theoretical assumption is, for instance, conceptualized in the compound-cue model (Logan & Bundesen, 2003; Schneider & Logan, 2007). According to this model, in every trial, the cue and the stimulus are encoded and form a compound that is used to retrieve the correct response from memory. When the cue repeats, the cue encoding process is primed, resulting in faster responses than in cue switches, where cue encoding has to proceed without the help of priming. Thus, this model explains the task-switch cost assessed with the 1:1 mapping of cues to task pairs without any need to switch task pairs.

Models that are based on the finding that cued task-switching produces both cue-switch costs and “pure” task-switch cost propose the involvement of two processes, including cue-related processes and task-related processes. Mayr und Kliegl (2003) argue, for example, that cue-switch costs reflect cue-encoding processes that result in the activation of a task-set in memory and that are facilitated due to priming when a cue repeats. The “pure” task-switch cost, in contrast, is assumed to reflect the implementation of an attentional configuration that is appropriate with the retrieved task set.

In Experiment 1, we sought to examine how cue-switch costs and “pure” task-pair switch costs contribute to the performance differences between task-pair switches and task-pair repetitions observed in studies with one cue per task pair. We hypothesized to observe, in addition to cue-switch costs, substantial “pure” task-pair switch costs, providing evidence against the view that task-pair switching is solely based on cue priming and does not require the switching between task-pair sets. Moreover, we predicted the PRP effect at the local level of dual-task processing.

### Method

#### Participants

Twenty-four subjects (20 women; 22 right-handed; *M*_age_ = 20.6 years; *SD* = 2.7) participated in return for partial course credit and gave informed consent. All participants had normal or corrected-to-normal vision and no hearing impairments.

#### Stimuli, tasks, and responses

We employed the same cues as Altmann (2006) did in his task-switching study, and we adopted the stimuli, tasks, and responses from Hirsch et al. (2018, Experiment 2). As cues, we used the letters *H*, *U*, *L,* and *W*. Two tones (200 Hz and 600 Hz) served as S1, whereas eight pictures (7 cm × 6 cm) served as S2. The cues were displayed in 28-point black Arial font and appeared, like the pictures, in the center of a white 17-inch screen. The pictures showed either a black can (i.e., teapot) or a black cup, with upright or upside-down orientation, and with a handle on the left or the right side of the object.

T1 was a tone discrimination task. Subjects were instructed to respond to the low-pitch tone by pressing the *Y* key of a QWERTZ keyboard with the middle finger of their left hand and to the high-pitch tone by pressing the *X* key with the index finger of their left hand. Considering the spatial–musical association of the response code’s effect (e.g., Keller & Koch, 2006, 2008; Rusconi, Kwan, Giordano, Umiltà, & Butterworth, 2006), the stimulus–response mapping was not counterbalanced across subjects.

T2 was a spatial orientation task (i.e., to decide whether the handle was on the left or right side of the object) or an object identity task (i.e., to decide whether the object was a can or a cup). Subjects pressed the *N* key with the index finger of the right hand when the handle was on the left side of the object or when the object was a can. The *M* key was pressed with the middle finger of the right hand when the handle was on the right side of the object or when the object was a cup. Taking into account the spatial stimulus–response compatibility effect (e.g., Fitts & Deininger, 1954; Fitts & Seeger, 1953), the stimulus–response mapping was not counterbalanced across subjects for the handle task. For the object task, we counterbalanced the response keys across subjects.

The tasks were combined to two task pairs with a constant T1 and a varying T2. Task-Pair 1 comprised the tone discrimination task as T1 and the handle task as T2, whereas Task-Pair 2 included the object task as T2. The cues *H* and *U* were used for Task-Pair 1 and the cues *L* and *W* for Task-Pair 2, counterbalanced across subjects.

#### Procedure

The experiment was run in a single session with one subject at a time. Each session began with the presentation of the instructions on the monitor. The instructions emphasized speed and accuracy for both tasks. Then, one practice block of 18 trials, followed by six experimental blocks, each consisting of 48 trials, were presented. Experimental trials were preceded in each block by one nonrecorded warm-up trials. In each trial, we first presented a task-pair cue for 600 ms, followed by S1 for 100 ms. After a random SOA of 50 ms or 800 ms, S2 was presented for 100 ms. The trials were separated by an intertrial interval (i.e., time interval between the response for S2 in trial *n* − 1 and the presentation of the task-pair cue in trial *n*) of 1,000 ms. The presentation of the task-pair sequences was random, thereby resulting in an almost even distribution of task-pair switches with cue switches (33.3%), task-pair switches with cue repetitions (33.0%), and task-pair repetitions with cue repetitions (33.7%).

#### Design

Performance in T1 and T2 was analyzed based on a 2 × 3 repeated-measures design with the within-subjects independent variables SOA (50 ms vs. 800 ms) and sequence (cue repetition trials with a task-pair repetition, cue-switch trials with a task-pair repetition, vs. cue-switch trials with a task-pair switch). For the cue-switch costs contrast, we compared cue-switch trials and cue-repetition trials. Note that both trial types were associated with a task-pair repetition. For the task-pair switch-cost contrast, we compared task-pair switches and task-pair repetitions, both of which came along with a cue switch. The dependent variables were reaction times (i.e., RT) and error rates.^1^

### Results

**Fig. 1:**
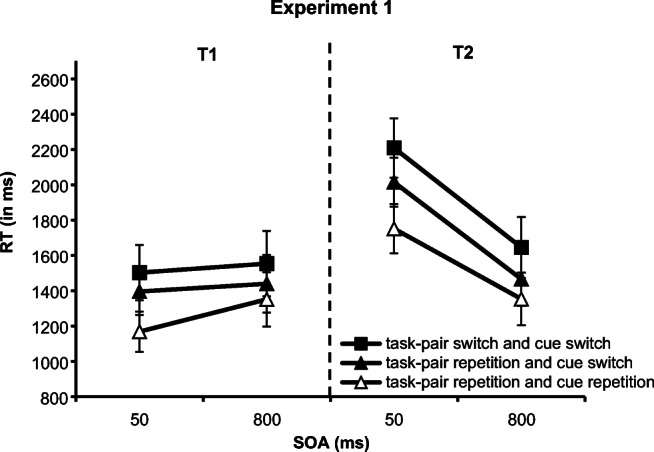
RT (in ms) in Experiment 1 for Task 1 (T1) and Task 2 (T2) as a function of task-pair sequence (task-pair switch with cue switch, task-pair switch with cue repetition, vs. task-pair repetition with cue repetition) and stimulus onset asynchrony (SOA; 50 ms vs. 800 ms). Error bars represent the standard error of the mean

Practice blocks, warm-up trials, trials following an error in T1 and/or T2, and trials with RTs deviating more than three standard deviations from each participant’s mean RT per condition (T1: 1.59%; T2: 1.64%) were discarded from both the RT analysis and the error analysis. In contrast to the error analysis, for the RT analysis, we also excluded trials with an erroneous response in T1 and/or T2. We ran separate analyses of variance (ANOVAs) on mean RTs and error rates (see Fig. 1 and Table 2).

**Table 2:** Mean error rates (in percentage; standard errors in parenthesis) in Experiment 1 for Task 1 (T1) and Task 2 (T2) as a function of task-pair sequence (task-pair switch with cue switch, task-pair repetition with cue switch, vs. task-pair repetition with cue repetition) and stimulus onset asynchrony (SOA; 50 ms vs. 800 ms)

	**T1**			**T2**		
	SOA 50 ms	SOA 800 ms	SOA effect	SOA 50ms	SOA 800 ms	SOA effect
Task-pair switch and cue switch	3.8 (0.9)	3.0 (0.5)	0.8	12.9 (1.6)	9.4 (1.7)	3.5
Task-pair repetition and cue switch	3.8 (0.7)	4.3 (0.8)	−0.5	9.7 (1.3)	8.3 (1.3)	1.4
Task-pair repetition and cue repetition	4.3 (0.8)	3.6 (0.8)	1.2	8.6 (0.9)	7.7 (1.1)	0.9
Task-pair switch costs	0	−1.3		3.2	1.1	
Cue switch costs	−0.5	0.7		1.1	0.6	

### Cue-switching contrast

#### T1

For RT1, there was a main effect of sequence *F*(1, 23) = 34.628, *p* < .001, η_p_^2^ = .601. Subjects responded more slowly in cue switch trials than in cue repetition trials (1,418 ms vs. 1,260 ms), reflecting cue switch costs of 158 ms. The main effect of SOA was significant, too, *F*(1, 23) = 6.895, *p* = .015, η_p_^2^ = .231. Responses were slower with long than with short SOA (1,396 ms vs. 1,283 ms; i.e., reversed SOA effect in T1). Finally, the interaction of sequence and SOA was significant, *F*(1, 23) = 22.730, *p* < .001, η_p_^2^ = .497, indicating a larger reversed SOA effect in cue-repetition trials than in cue-switch trials (−183 ms vs. −44 ms). Post hoc two-tailed *t* tests showed that the SOA effect in cue switches was not significant, *t*(23) = 1.045, *p* = .307, whereas the reversed SOA effect in cue repetitions was significant, *t*(23) = 3.726, *p* = .001.

For the error rates, the main effects of sequence and SOA, both *F*s < 1 and *p*s > .878, were not significant. Moreover, the sequence by SOA interaction was not significant, *F*(1, 23) = 1.201, *p* = .284, η_p_^2^ = .05.

#### T2

For RT2, the ANOVA yielded the main effects of sequence, *F*(1, 23) = 25.351, *p* < .001, η_p_^2^ = .524, and SOA, *F*(1, 23) = 137.957, *p* < .001, η_p_^2^ = .857. Responses were slower in cue-switch trials than in cue-repetition trials (1,741 ms vs. 1,553 ms) and with short than long SOA (1,884 ms vs. 1,411 ms), resulting in cue switch costs of 188 ms and a PRP effect of 473 ms. The interaction of sequence and SOA was significant, too, *F*(1, 23) = 19.568, *p* < .001, η_p_^2^ = .46. The PRP effect was larger in cue switch trials than cue repetition trials (548 ms vs. 398 ms).

For the error rates, the main effect of SOA, *F*(1, 23) = 2.718, *p* = .113, η_p_^2^ = .106, the main effect of sequence, *F*(1, 23) = 1.311, *p* = .264, η_p_^2^ = .054, and the interaction of SOA and sequence, *F* < 1, were not significant.

### Task-pair switching contrast

#### T1

The ANOVA on RT1 showed a main effect of sequence, *F*(1, 23) = 5.239, *p =* .032, η_p_^2^ = .186. Subjects responded more slowly in task-pair switch trials than in task-pair repetition trials (1,529 ms vs. 1,418 ms) leading to task-pair switch costs of 111 ms. The main effect of SOA, *F*(1, 23) = 1.362, *p =* .255, η_p_^2^ = .056, and the interaction of sequence and SOA were not significant, *F <* 1.

For the error analysis, neither the main effect of sequence nor the main effect of SOA, both *F*s < 1 and *p*s > .362, were significant. Moreover, the interaction of sequence and SOA was not significant, *F*(1, 23) = 1.888, *p =* .183, η_p_^2^ = .076.

#### T2

For RT2, there were significant main effects of sequence, *F*(1, 23) = 14.381, *p =* .001, η_p_^2^ = .385, and SOA, *F*(1, 23) = 231.381, *p < .*001, η_p_^2^ = .91. Responses were slower in task-pair switch trials than in task-pair repetition trials (1,928 ms vs. 1,741 ms) and with short than with long SOA (2,112 ms vs. 1,556 ms), reflecting task-pair switch costs of 187 ms and a PRP effect of 555 ms. The interaction of sequence and SOA was not significant, *F* < 1.

The ANOVA on the error rates showed a main effect of SOA with more erroneous responses with short than with long SOA (11.3% vs. 8.9%), and hence, a PRP effect of 2.4%, *F*(1, 23) = 5.103, *p =* .034, η_p_^2^ = .182. The main effect of sequence was significant, too, *F*(1, 23) = 5.103, *p =* .034, η_p_^2^ = .182. There were more erroneous responses in task-pair switch trials than in task-pair repetitions trials (11.2% vs. 9.0%), indicating task-pair switch costs of 2.2%. The interaction of sequence and SOA, *F* < 1, was nonsignificant.

### Discussion

At the local level of dual-task processing, we found a PRP effect reflecting T2 performance deteriorations induced by the requirement of temporally overlapping task-processing. At the global level, we observed, besides cue-switch cost which might reflect priming of cue encoding processes, “pure” task-pair switch costs that cannot be accounted for by cue switching. Consequently, the task-pair switch cost assessed with a 1:1 cue to task-pair mapping seems to reflect to a substantial degree cognitive processes involved in reconfiguring the cognitive system to perform a specific task pair. The existence of “pure” task-pair switch costs provides further evidence that the identity of T1 and T2 is jointly represented in a single mental representation.

Note that in the cue-switch contrast for RT in T1, we revealed a reversed SOA effect, meaning that T1 performance was worse with long than with short SOA. As can be seen in Fig. 1, it seems that the reversed SOA effect in T1 is mainly driven by the fast reactions in cue repetitions with short SOA. In addition to the main effect of SOA, there was an interaction between SOA and cue sequence, reflecting a larger reversed SOA effect in cue repetitions than in cue switches. Cue repetition trials are the only trials where the cue, the T1 type, and the T2 type of the previous trial are repeated. Thus, cue repetition trials represent a repetition of the whole previous trial episode. It might be that the onset of S2 (further) primes the episodic representation of the previously performed task-pair. With long SOA, where S2 is not presented in rapid succession to S1, the priming effect is attenuated and thus response times increase. This explanation has to be tested in future research.

## Experiment 2

After ruling out the alternative explanation in Experiment 1 that task-pair switch costs do not reflect solely cue-encoding benefits due to lower-level priming processes instead of task-pair set control, Experiment 2 aimed to investigate the point in time when task-pair sets are activated. Hirsch et al. (2018) discussed an episodic binding account (see Frings, Hommel, et al., 2020a; Frings, Koch, et al., 2020b, for reviews) and a hierarchical account. They favored the hierarchical account, but did not test it systematically.

According to an *episodic binding account*, a task-pair set is only activated after performing a dual task. This suggests that the processing of a dual task results in an episodic representation of the specific task-pair performed in the previous trial. Thus, task-pair switch costs would result from episodic aftereffects (i.e., inertia of previously formed task-pair set) and a task-pair set is formed only *after* performing a dual task rather than activated before performing a dual task.

In contrast, the *hierarchical account* assumes that the task-pair set is activated before performing a dual task, for instance, by a cue, and task-pair switch costs reflect proactive dual-task control. More specifically, the task-pair set is assumed to be an explicit (i.e., separate) representation that is organized at a hierarchically higher level than the subtask-specific task sets of T1 and T2 (for similar idea concerning multistep sequential tasks, see Cooper & Shallice, 2000; Humphreys & Forde, 1998). The rationale behind this notion is that the identity of T1 and T2 has to be available before processing T1 and T2. The hierarchy is defined by the temporal precedence of task-pair set activation that allows the identification of the identity of T1 and T2, and hence the subsequent selection of the T1 and T2 task sets.

To test these accounts, we combined the task-pair switching logic with a go/no-go-like manipulation in Experiment 2 (see, e.g., Koch & Philipp, 2005; Lenartowicz, Yeung, & Cohen, 2011; Schuch & Koch, 2003, for go/no-go manipulations in the task-switching domain). According to our go/no-go procedure, at the beginning of each trial, subjects are presented with a task-pair cue allowing for the activation of the relevant task-pair set before the onset of the imperative S1 and S2 (i.e., advanced preparation). In go trials, subjects execute the responses for T1 and T2 and hence complete the processing of the task pair. In contrast, in no-go trials, the task-pair cue is not followed by the presentation of S1 and S2. Thus, subjects execute neither the T1 response nor the T2 response. Such trials with a task cue but no task stimuli are also referred to as “cue-only trials” (e.g., Lenartowicz et al., 2011). Since it is not predictable whether a go trial or no-go trial is presented, subjects have to prepare for the next task pair in both trial types.

The episodic binding account predicts an absence of task-pair switch costs after cue-only trials. This is because according to this account, a task-pair set is only activated after the processing of a task pair, and in cue-only trials, subjects prepare for a task pair but do not perform it. In contrast, the hierarchical account hypothesized the existence of task-pair switch costs after cue-only trials. This is because according to this account, a task-pair set has to be available before starting to perform a dual task. When in cue-only trials, the task-pair set is activated based on the task-pair cue, and its activation persists into the next trial even if the task-pair is not performed, task-pair switch costs should occur after cue-only trials.

To study task-pair set activation, we conducted Experiment 2. We predicted performance to be better when the preparation interval (i.e., cue-stimulus interval; CSI) for the upcoming task pair is long rather than short. We also hypothesized finding task-pair switch costs and a reduction of these costs with long CSI relative to short CSI. Following Hirsch et al. (2018), task-pair switch costs were predicted to occur after both go trials and cue-only trials, providing evidence for the hierarchical account.

In previous studies, the reduction of switch costs with long CSI in go trials has been used as a manipulation check for the notion that subjects employed the cue for advance preparation (e.g., Schuch & Koch, 2003). However, since it has been shown that CSI variations have no substantial effect on performance in single-task conditions, it is assumed that in mixed-task conditions, differences in overall performance with short and long CSIs do not reflect unspecific temporal preparation but rather task-specific preparation, which does not have to be switch specific (i.e., task repetitions in mixed-task conditions have to be prepared as indicated by mixing costs; Koch & Philipp, 2005). Therefore, in addition to a preparatory reduction of task-pair switch costs (i.e., switch-specific preparation), differences in the overall performance across CSIs (i.e., task-specific preparation) can be used as a manipulation check for the notion that subjects use the cue for preparing the next task-pair.

Note that the predicted existence of task-pair switch costs after cue-only trials does not indicate that task-pair sets are only activated prior to task execution and that episodic representations play no role in task-pair switching. This is because the hierarchical account and the episodic binding account are not mutually exclusive. From an integrative view, it is possible that task-pair set activation starts prior to task execution (i.e., after cue presentation) but its completion requires the onset of the target stimuli (cf. residual switch costs in the general task-switching domain; e.g., Nieuwenhuis & Monsell, 2002). Further, it is conceivable that in addition to task-set activation prior to task-pair execution, an episodic representation of what exactly has happened in the trial is created after the completion of a task pair. Consequently, the use of a go/no-go-like variation enables us to examine whether a task-pair set is activated, at least partly, prior task-pair execution, but it does not allow us to explore whether the creation of an episodic representation of the previous trial contributes, in addition to the task-pair set activation prior to the dual-task execution, to task-pair switch costs.

Note that we did not vary the temporal overlap in T1 and T2 processing in Experiment 2. This is because we had no specific hypothesis about how the temporal overlap in task processing could affect the emergence of task-pair switch costs. Without such hypothesis, an experimental design with even more independent variables would have had made the interpretation of interaction effects more complicated.

Moreover, we used only one cue per task pair in Experiment 2 to ensure the comparability with previous studies. However, since in the task-switching domain, Swainson, Martin, and Prosser (2017) and Swainson, Prosser, Karasilev, and Romanczuk (2019) found significant task switch costs after cue-only trials even when controlling for cue switching (see Brass and von Cramon, 2004, for comparable results employing a different experimental design), we argue that switching both cues and task pairs will substantially contribute to task-pair switch costs (if existent) in Experiment 2.

### Method

#### Participants

A new group of twenty-four subjects with normal or corrected-to normal vision and intact hearing ability (17 women; 21 right-handed; *M*_age_ = 22.7 years; *SD* = 3.7) participated in the experiment. We excluded data of one participant from all analyses due to an excessive error rate (i.e., 64% errors) and tested one additional subject to replace this data set (i.e., leaving 24 data sets for the analyses). All participants gave informed consent.

#### Stimuli, tasks, responses, and procedure

The stimuli, tasks, and responses were identical to those used in Experiment 1. To ensure the comparability with previous task-pair switching studies (Hirsch et al., 2017, 2018), as opposed to Experiment 1, we used the German words for *side* and *object* (i.e., “Seite” and “Objekt”) as cues for the two task pairs.

#### Procedure

The experiment was run in a single session with one subject at a time. As in Experiment 1, the instructions were displayed at the monitor and emphasized speed and accuracy for both tasks. The experiment started with two practice blocks, consisting of 16 trials each. The first practice block had only go trials, and the second block included both go trials and cue-only trials. Then nine experimental blocks with 64 trials each were presented. Each experimental block was preceded by a warm-up trial and contained 75% go trials and 25% cue-only trials.

At the start of each go trial, a cue was presented for 250 ms, followed by a blank screen for 100 ms or 1,000 ms. After this CSI of either 350 ms or 1250 ms, S1 was presented for 100 ms, and after a SOA of 50 ms, S2 was displayed for 100 ms. To hold the response–stimulus interval (i.e., RSI, which is the time interval between the response for T2 in trial *n* − 1 and the presentation of S1 in trial *n*) constant at 1,850 ms across trials with a short and long CSI, the response-cue-interval (i.e., RCI) was 600 ms or 1,500 ms and was manipulated inversely to the CSI. The same trial procedure was used in cue-only trials, except that neither S1 nor S2 was presented between the cues of two consecutive trials. Thus, after the expiration of the CSI, the next trial started.

The task-pair sequence was presented randomly with the stipulation that in experimental trials, there was the same number of each combination of the task-pair sequence and the CSI. This applies to both go trials and cue-only trials. Cue-only trials were always followed by go trials.

#### Design

To explore the effect of cue-only trials on task-pair switch costs, we used a 2 × 2 × 2 repeated-measures design with the independent within-subjects variables task-pair sequence (task-pair switch vs. task-pair repetition), CSI (350 ms vs. 1,250 ms), and go condition in trial *n* − 1 (go trial vs. cue-only trial). Dependent variables were RT and error rates.

### Results

**Fig. 2:**
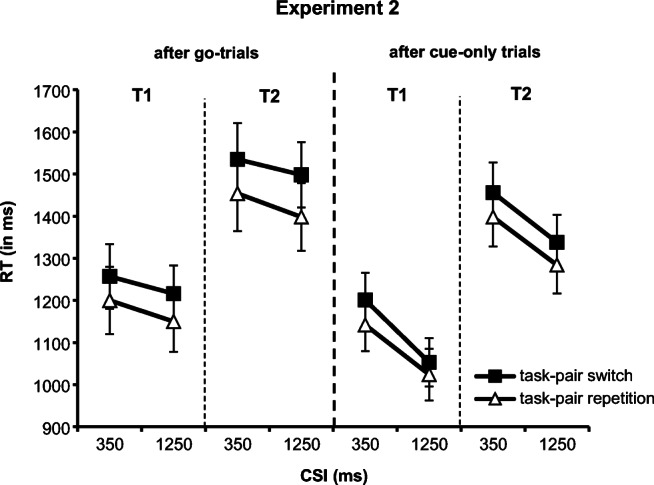
RT (in ms) in Experiment 2 after go trials and cue-only trials for Task 1 (T1) and Task 2 (T2) as a function of task-pair sequence (task-pair switch vs. task-pair repetition) and cue stimulus interval (CSI; 350 ms vs. 1,250 ms). Error bars represent the standard error of the mean

We used the same outlier criteria as in Experiment 1 (1.03% in T1, 1.01% in T2). Separate ANOVAs were run on mean RTs and error rates (see Fig. 2 and Table 3).

**Table 3:** Mean error rates (in percentage; standard errors in parenthesis) in Experiment 2 after go trials and cue-only trials for Task 1 (T1) and Task 2 (T2) as a function of task-pair sequence (task-pair repetition vs. task-pair switch) and cue-stimulus interval (CSI; 350 ms vs. 1,250 ms)

	T1			T2		
	CSI 350 ms	CSI 1250 ms	CSI effect	CSI 350ms	CSI 1250 ms	CSI effect
After go trials						
Task-pair switch	4.4 (0.9)	3.8 (0.8)	0.6	11.1 (1.6)	9.8 (1.1)	1.3
Task-pair repetition	5.0 (1.1)	4.1 (0.8)	1.1	6.6 (1.0)	7.0 (1.6)	−0.4
Task-pair switch costs	−0.6	−0.3		4.5	2.8	
After cue-only trials						
Task-pair switch	2.4 (0.6)	3.8 (0.8)	−1.4	7.3 (1.2)	6.8 (1.0)	0.5
Task-pair repetition	3.4 (0.8)	2.4 (0.8)	1.0	6.9 (1.0)	7.8 (1.2)	−0.9
Task-pair switch costs	−1.0	1.4		0.4	−1.0	

#### T1

For RT1, the ANOVA showed main effects of task-pair sequence, *F*(1, 23) = 22.813, *p <* .001, η_p_^2^ = .498, and CSI, *F*(1, 23) = 64.415, *p <* .001, η_p_^2^ = .737. RTs were higher in task-pair switch trials than task-pair repetition trials (1,181 ms vs. 1,129 ms) and with short than long CSI (1,200 ms vs. 1,110 ms), reflecting task-pair switch costs of 52 ms and a CSI effect of 90 ms. Moreover, there was a significant main effect of go condition in trial *n* − 1, with slower responses after go trials than cue-only trials (1,206 ms vs. 1,105 ms), *F*(1, 23) = 24.906, *p <* .001, η_p_^2^ = .52. The interaction of go condition in trial *n* − 1 and CSI was significant, too, *F*(1, 23) = 9.762, *p =* .005, η_p_^2^ = .298. The CSI effect was larger after cue-only trials than after go trials (133 ms vs. 55 ms). As indicated by post hoc two-tailed *t* tests, the CSI effect was significant after both cue-only trials, *t*(23) = 2.424, *p* = .024, *d* = 0.495, and go trials, *t*(23) = 7.885, *p* < .001, *d* = 1.609. Importantly, the interaction of task-pair sequence and go condition in trial *n* − 1 was nonsignificant, *F*(1, 23) = 1.538, *p =* .227, η_p_^2^ = .063, indicating that task-pair switch costs did not differ significantly in their size depending on whether they occurred after go trials or cue-only trials (61 ms vs. 44 ms). The two-way interaction of CSI and task-pair sequence and the three-way interaction of CSI, task-pair sequence, and go-condition in trial *n* − 1 were not significant, either, both *F*s < 1.

The ANOVA on the error rates yielded a significant main effect of go condition in trial *n* – 1, with more erroneous responses after go trials than cue-only trials (4.3% vs. 3%), *F*(1, 23) = 7.047, *p =* .014, η_p_^2^ = .235. Like all remaining effects, all *F*s < 1.773 and *p*s > .196, the interaction of CSI and task-pair sequence, *F*(1, 23) = 2.264, *p =* .146, η_p_^2^ = .09, and the interaction of task-pair sequence and go condition in trial *n* − 1 were nonsignificant as well, *F* < 1.

#### T2

For RT2, there were significant main effects of task-pair sequence, *F*(1, 23) = 37.221, *p <* .001, η_p_^2^ = .618, and CSI, *F*(1, 23) = 56.284, *p <* .001, η_p_^2^ = .71, reflecting slower responses in task-pair switch trials than in task-pair repetition trials (1,456 ms vs. 1,384 ms) and with short than long CSI (1,461 ms vs. 1,380 ms). Thus, there were task-pair switch costs of 72 ms and a CSI effect of 81 ms. There was also a main effect of go condition in trial *n* – 1, with higher RTs after go trials than cue-only trials (1,472 ms vs. 1,368 ms), *F*(1, 23) = 25.506, *p <* .001, η_p_^2^ = .526. The interaction of CSI and go condition in trial *n* − 1 was significant, too, *F*(1, 23) = 4.763, *p =* .04, η_p_^2^ = .172. Like for RT1, the CSI effect was more pronounced after cue-only trials than go trials (115 ms vs. 47 ms). The interaction of task-pair sequence and go condition in trial *n* − 1 was not significant, *F*(1, 23) = 4.202, *p =* .052, η_p_^2^ = .154. However, there was a trend towards smaller task-pair switch costs after cue-only trials than go trials (55 ms vs. 91 ms). As indicated by post hoc two-tailed *t* tests the task-pair switch cost was significant after both go trials, *t*(23) = 5.425, *p* < .001, *d* = 1.099, and cue-only trials, *t*(23) = 4.335, *p* < .001, *d* = 0.896. The interaction of CSI and task-pair sequence and of CSI, task-pair sequence, and go-condition in trial *n* − 1 were nonsignificant, both *F*s < 1.

For the error rates, the ANOVA showed a main effect of task-pair sequence, *F*(1, 23) = 8.201, *p =* .009, η_p_^2^ = .263. There were more errors in task-pair switch trials than in task-pair repetition trials (8.8% vs. 7.1%), resulting in task-pair switch costs of 1.7%. The interaction of task-pair sequence and go condition in trial *n* − 1 was significant, too, *F*(1, 23) = 9.535, *p =* .005, η_p_^2^ = .293. Task-pair switch costs were smaller after cue-only trials than go trials (−0.2% vs. 3.6%), and post hoc two-tailed *t* test showed that task-pair switch costs were significant after go trials, *t*(23) = 4.819, *p* < .001, *d* = 0.58, and nonsignificant after cue-only trials, *t*(23) = 0.281, *p* = .781. Moreover, there was a numerical trend towards more error-prone responses after go trials than cue-only trials (8.6% vs. 7.2%). The main effect of go condition in trial *n* − 1, however, was nonsignificant, *F*(1, 23) = 3.587, *p =* .071, η_p_^2^ = .135. The interaction of CSI and task-pair sequence, *F*(1, 23) = 1.341, *p =* .259, η_p_^2^ = .055, and all remaining effects were nonsignificant as well, all *F*s < 1.

### Discussion

In line with our hypotheses, we observed task-pair switch costs in T1 which occur after both go trials and cue-only trials, providing evidence that task-pair sets are activated, at least partly, prior to the execution of a dual task. Moreover, overall performance was better with long CSI than with short CSI. The task-pair switch cost, however, was not affected by the CSI.

## General discussion

The objective of the present study was twofold. First, we aimed to assess cue switching as an alternative explanation for task-pair switch costs which are interpreted as evidence that the T1 and T2 identities are jointly represented in a single task-pair set. Second, we sought to investigate the point in time when task-pair sets are activated. More specifically, we tested whether a task-pair set might have to be activated *before* starting to perform a dual task, or whether the task-pair set might be formed as an episodic representation of the previous trial, and hence, be only available *after* performing a dual task. To this end, we ran two experiments. In Experiment 1, we used two cues per task pair and analyzed task-pair switching independently of cue switching. In Experiment 2, we employed a go/no-go-like variation and investigated the effects of cue-only trials on task-pair switch costs.

In Experiment 1, we observed, besides a PRP effect, cue switch costs in T1 and T2. Notably, there were also substantial “pure” task-pair switch costs in T1 and T2. In Experiment 2, we replicated the task-pair switch cost. Whereas in T1, task-pair switch costs did not significantly differ across cue-only trials and go trials, task-pair switch costs were reduced after cue-only trials in T2 (i.e., for error rates). Moreover, overall performance was improved after a long CSI compared with short CSI, and this CSI effect was larger after cue-only trials than after go trials. Task-pair switch costs, however, did not differ across CSIs.

### Cognitive processing at the local level of T1 and T2

At the local level of dual-task processing, we observed the expected PRP effect, indicating T2 performance deteriorations due to temporally overlapping task processing. This observation is consistent with numerous studies on SOA effects in dual-task performance (see e.g., Koch et al., 2018, for a review).

### Cognitive processing at the global level of task pairs

In the present study, we observed substantial task-pair switch costs in T1 and T2 with abstract cues (Experiment 1) and CSIs of short and long duration (Experiment 2). Given that previous task-pair switching studies used exclusively words as cues and an intermediate CSI (e.g., Hirsch et al., 2017, 2018), the replication of task-pair switch costs in the present study shows that this performance cost represents a reliable effect.

#### The role of cue switching

In Experiment 1, task-pair switch costs in T1 occur even when controlling for cue switching (see Forstmann, Brass, & Koch, 2007, for a discussion on methodological issues and interpretational problems associated with the 2:1 mapping). This is an important finding because it rules out the alternative explanation that task-pair switch costs assessed with the 1:1 cue to task pairs mapping exclusively reflect performance costs arising from switching cues rather than from switching task pairs. However, we also observed cue switch costs when the task-pair remained unchanged, indicating that in previous studies using a 1:1 mapping of cues to task pairs, both cue switching and task-pair switching may have contributed to the observed task-pair switch cost. Even though task-pair switch costs were smaller than cue switch costs in Experiment 1, switching task pairs per se produced a substantial cost that cannot be accounted for by cue switching itself.

Following the model proposed by Mayr and Kliegl (2003), the finding of cue-switch costs and “pure” task-pair switch costs suggests the involvement of two processes in task-pair switching situations. Cue-switch cost might reflect the cue-driven retrieval of task-pair sets from memory, whereas the “pure” task-pair switch cost might indicate that the activated task-pair set is used to implement an attentional configuration suitable for correctly performing a specific task pair. Consequently, the findings of Experiment 1 suggest that task-pair switch-costs measure aspects of task-pair set control instead of solely reflecting cue-encoding benefits due to lower-level priming processes.

#### Activation of task-pair sets

In Experiment 2, we found task-pair switch costs after cue-only trials. In cue-only trials, subjects were presented with a task-pair cue, but neither with S1 nor with S2. Compatible with a hierarchical account of task-pair set activation, this finding indicates that subjects used the cue to activate (at least partly) the appropriate task-pair set and that the task-pair set activation persisted into the next trial, irrespective of whether the corresponding task-pair was performed or not, leading to task-pair switch costs. Note, however, that this finding does not rule out the possibility that in addition to the task-pair set activation prior to dual-task execution, an episodic representation of the previously performed task pair is created after the completion of a dual task in go trials.

A further finding of Experiment 2 was that there was an improvement in overall performance with long CSI compared with short CSI, but task-pair switch costs were not affected by the CSI. Since the RSI was held constant in Experiment 2, the beneficial effect of the long CSI on overall performance cannot be due to differences in the time available between two task pairs, rather the effect seems to be specific to the preparation time provided by the CSI. One possible explanation for the lack of a preparatory reduction of task-pair switch costs (i.e., switch-specific preparation) is that even the short CSI of 350 ms might have been already too long and allows the task-pair set in both task-pair switch and repetition trials to be strongly activated.^2^ In fact, studies on preparation at the global level of dual-task processing that observed a reduction of performance costs related to order switching with long CSI used considerably shorter CSIs of 150 ms.

Note though that Koch and Philipp (2005) showed that the effect of CSI in single-task conditions was very small (10–20 ms), suggesting that the substantial CSI main effect of about 90 ms in the present Experiment 2 reflects task-specific preparation in mixed task-pair conditions, reducing the interference that is, for example, reflected in task-mixing costs (see Kiesel et al., 2010, for a discussion). Hence, considering that the CSIs were rather long and cue-only trials were rare in Experiment 2, we assume that the participants used the cue for preparing the upcoming task in both cue-only trials and go trials.

Importantly, whereas task-pair switch costs in T1 did not differ depending on whether the task-pair was performed or not in the previous trial, task-pair switch costs in T2 were reduced after cue-only trials (i.e., for error rates). Even though within the scope of the task-pair switching logic the focus lies on performance in T1, it should be noted that the finding of reduced task-pair switch costs in T2 does not refute the hierarchical account because the task-pair switch costs was still substantial. It might, however, indicate that the episodic representation of the specific task-pair performed in the previous trial might have an additional impact on task-pair switching performance.

Note that we used a 1:1 mapping of cues to task pairs in Experiment 2, to ensure the comparability with previous task-pair switching studies. Since in the task-switching domain, significant task-switch costs (i.e., “pure” switch costs) have been found after cue-only trials even when controlling for cue switching (e.g., Swainson et al., 2017; Swainson et al., 2019; see also Brass and von Cramon, 2004), we assume that switching both cues and task pairs substantially contributed to the task-pair switch costs observed after go trials and cue-only trials in Experiment 2. However, we cannot draw strong conclusions on the exact extent to which cue switch costs and “pure” switch costs contributed to the task-pair switch cost after cue-only trials.

### Summary and conclusion

Task-pair switch costs do not merely reflect cue-switch costs, but switching task pairs per se produces an additional performance cost independently of cue switching. Consequently, task-pair switch costs can be interpreted as providing strong evidence for task-pair sets. Since task-pair switch costs occur even when subjects prepare for a task-pair but do not perform it, task-pair sets seem to be activated, at least partly, before the processing of a task-pair. Thus, task-pair switch costs seem to be the result of proactive dual-task control. How inertia of the previously formed task-pair set contributes to task-pair switch costs has to be examined in future studies.

These findings suggest that the multicomponent mental representation of a dual task proposed by Hirsch et al. (2017, 2018) is hierarchically organized. In this representation, the task-pair set is organized at a hierarchically higher level than the subtask-specific task sets. This is because the task-pair set allows the identification of the T1 and T2 identity and therefore has to be activated before the subtask-specific task sets of T1 and T2 are activated.

#### Open practices statement

The data sets (i.e., raw trial-by-trial data without demographic information about the participants) generated during the current study are available in the PsychArchives, (10.23668/psycharchives.3140). None of the experiments was preregistered.

## Footnotes

1 Note that we did not calculate a priori statistical power analyses for the experiments of the present study. However, for our first task-pair switching study (Hirsch et al., 2017), we ran such an analysis and decided to test 24 participants (i.e., when considering counterbalancing constraints). Since then we have used this sample size for all our task-pair switching experiments (e.g., Hirsch et al., 2017, 2018) and have observed task-pair switch costs across all these experiments. By testing 24 participants in the present experiments, we thus selected a plausible sample size.

2 To rule out the possibility that the observed task-pair switch costs in T1 after no-go trials were due to the sequence defined by the task-pair before the no-go trials (i.e., task-pair in trial *n* − 2), we repeated all analyses with the within-subjects independent variable *n* − 2 sequence (e.g., *n* − 2 task-pair switch trials with Task-Pair 1 in the go-condition of trial *n* − 2, followed by the cue-only condition in trial *n* − 1, and Task-Pair 2 in the go condition of trial *n* vs. *n* − 2 task-pair repetition trial with Task-Pair 2 in the go condition of trial *n* − 2, followed by the cue-only condition in trial *n* − 1, and Task-Pair 2 in the go condition of trial *n*) instead of *n* − 1 sequence (e.g., *n* − 1 task-pair switch trial with Task-Pair 1 in the cue-only condition of trial *n* − 1 and Task-Pair 2 in the go condition of trial *n* vs. *n* − 1 task-pair repetition trial with Task-Pair 2 in the cue-only condition of trial *n* − 1 and the go-condition of trial *n*). However, all effects including the *n* − 2 sequence were non-significant, main effect *n* − 2 task-pair sequence: RT1 with *F*(1, 23) = 1.273, *p* = .271, η_p_^2^ = .052, error rates in T1 with *F* < 1, RT2 with *F*(1, 23) = 2.304, *p* = .143, η_p_^2^ = .091, error rates in T2 with *F*(1, 23) = 1.150, *p* = .295, η_p_^2^ = .048; interaction go condition and *n* − 2 sequence: RT1 with *F*(1, 23) = 1.333, *p* = .26, η_p_^2^ = .055, error rates in T1 with *F*(1, 23) = 2.341, *p* = .14, η_p_^2^ = .092, RT2 with *F* < 1, error rates in T2 with *F* < 1.
